# Hydrocephalus As Possible Prodromal Manifestation of COVID-19: A Report of Two Cases

**DOI:** 10.7759/cureus.34371

**Published:** 2023-01-30

**Authors:** Giovanni Torelli, Rocco Severino, Chiara Caggiano, Matteo Torelli, Luca de Martino, Giuseppe Russo

**Affiliations:** 1 Neurosurgery, Azienda Ospedaliera Universitaria (AOU) San Giovanni e Ruggi d'Aragona, Salerno, ITA; 2 Neurosurgery, Azienda Ospedaliera di Rilievo Nazionale Antonio Cardarelli, Naples, ITA; 3 Pharmacy, Federico II University, Naples, ITA

**Keywords:** ace-2 receptor, altered csf dynamics, neuro-inflammation, covid-19, hydrocephalus

## Abstract

Although the etiopathology of normal pressure hydrocephalus (NPH) is still not completely defined, several studies in recent years have highlighted the role of neuro-inflammation mediators in its development. During COVID-19, the infected host develops a multifaceted inflammatory syndrome, that may lead to an uncontrolled immune system response also localized in the host nervous system. In fact, the target of the viral Spike protein, the angiotensin-converting enzyme 2 (ACE2) receptors, is widely expressed in different areas of CNS such as the olfactory epithelium, and the choroid plexus. As for idiopathic NPH, the massive release of inflammatory mediators may result in altered CSF dynamics and consequent sudden clinical decompensation.

We report the cases of two patients with a known iNPH condition, in which neurological symptoms suddenly worsened, requiring hospitalization, without any evident precipitating cause. Both patients tested positive for the COVID-19 virus shortly after the neurological impairment, which had occurred, therefore, during the incubation period of the infection.

On the basis of our experience we advise, in cases of NPH patients with sudden neurological worsening, to perform a molecular COVID-19 swab at the moment of clinical impairment. We, therefore, recommend considering SARS-CoV-2 infection in the differential diagnosis of a sudden and otherwise unexplainable impairment of hydrocephalic patients. Furthermore, we believe clinicians should invite NPH patients to adopt adequate preventive measures to protect them from SARS-CoV-2 infection.

## Introduction

Normal pressure hydrocephalus (NPH) is defined as the presence of a dilated ventricular system with concomitant specific neurological signs (gait disorders, urinary incontinence, and cognitive decline) without intracranial hypertension [1]. When caused by other diseases (e.g., trauma, sub-arachnoidal hemorrhage, neoplasms, infections) NPH is defined as secondary (sNPH) [2], to distinguish it from a more frequent idiopathic form (iNPH) in which no other etiology can be recognized [3]. Despite NPH has been first described in the 1960s [4], its pathophysiology has been only partly explained. In particular, in recent years several studies have highlighted the role of neuro-inflammation in the genesis of NPH, both in the idiopathic [5-8] and in post-hemorrhagic secondary forms [9,10].

Since the beginning of the SARS-CoV-2 pandemic only three cases of previously healthy patients who developed NPH after COVID-19 infection have been described [11-13], suggesting that NPH may have been a complication of the neuro-inflammation response triggered by SARS-CoV2 in the nervous system.

In our article we report the cases of two patients with a known iNPH condition, in which neurological symptoms suddenly worsened, requiring hospitalization, without any evident precipitating cause. Both patients tested positive for the COVID-19 virus shortly after the neurological impairment, which had occurred, thereby, during the incubation period of the infection. We, therefore, suggest that COVID-19 infection in its prodromal phase should be considered in the differential diagnosis in cases of NPH patients with sudden and otherwise unexplained neurological decline.

## Case presentation

Case 1

**Figure 1:**
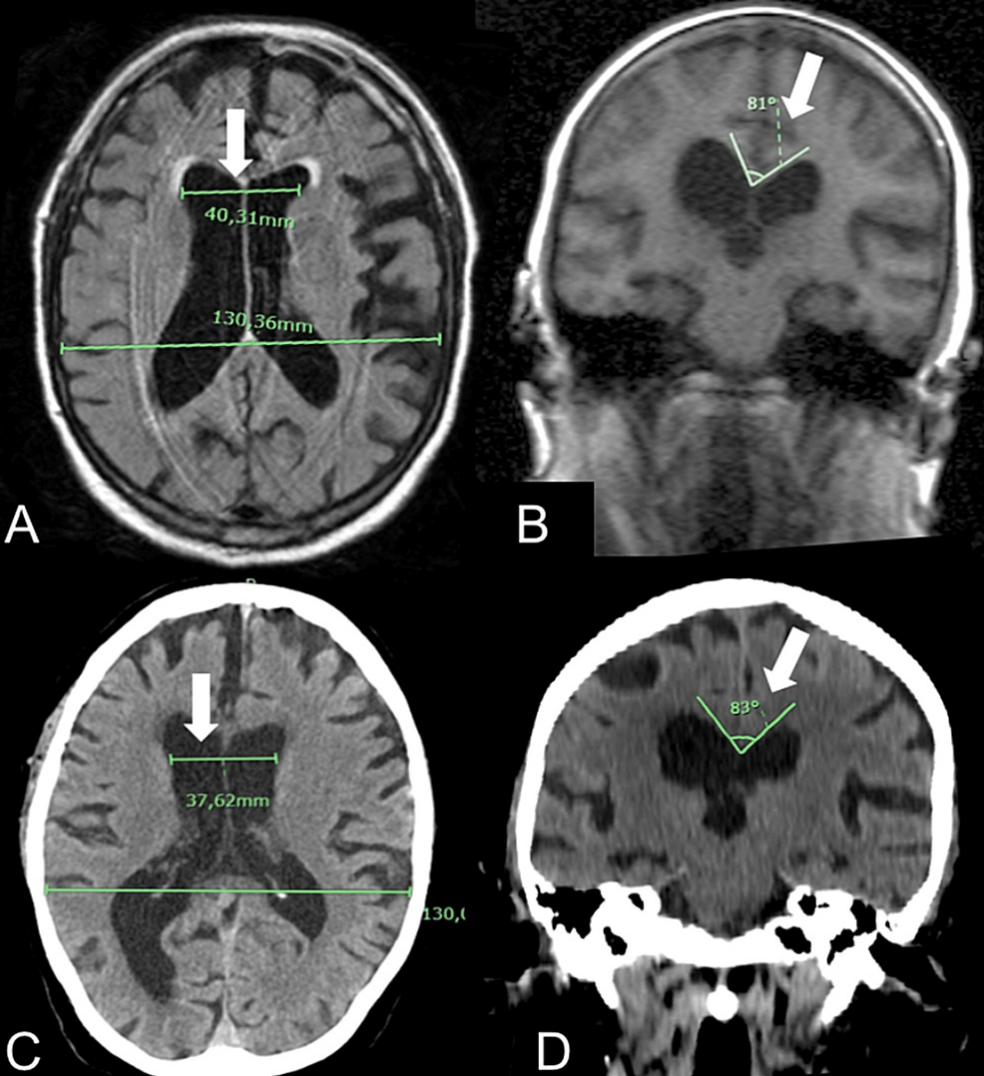
Radiological findings of Case 1 (A, B) Axial and coronal MRI, performed by the patient five months before the hospitalization, showing ventriculomegaly with features of NPH. (C, D) CT scan, performed at the time of neurological deterioration with negative COVID test, showing no substantial modifications of the ventricular system volume. The white arrows highlight the common features of NPH: Evans Index (A, C)>0.28; 0.31; callosal angle (B, D) < 100°.

A 79-year-old unvaccinated man presented with a history of progressive gait instability in the previous months and cognitive decline. He performed a brain MRI almost five months before the hospitalization, showing ventriculomegaly with NPH features (Figures 1A, 1B). He was admitted to our department complaining of a recent impairment of gait difficulties and an episode of loss of consciousness, with a negative molecular COVID-19 test. General examination was unremarkable, while the neurological examination was consistent with an NPH condition, showing mild ideo-motor slowdown and magnetic gait. A head CT scan showed an enlarged ventricular system with no evidence of CSF flow obstructions or signs of acute intracranial hypertension. After 24 hours the patient presented a sudden worsening of the neurological status with walking inability for severe gait instability. A new CT scan showed no variations in the size of the ventricular system nor other evident causes of the clinical deterioration (Figures 1C, 1D). A new COVID-19 molecular test, performed the following day for the onset of hyperpyrexia, resulted positive. The patient was then transferred to the COVID-19 area of our hospital where he gradually recovered. Once tested negative he returned to our department. The proposed ventriculoperitoneal shunting procedure was refused by the patient.

Case 2

**Figure 2:**
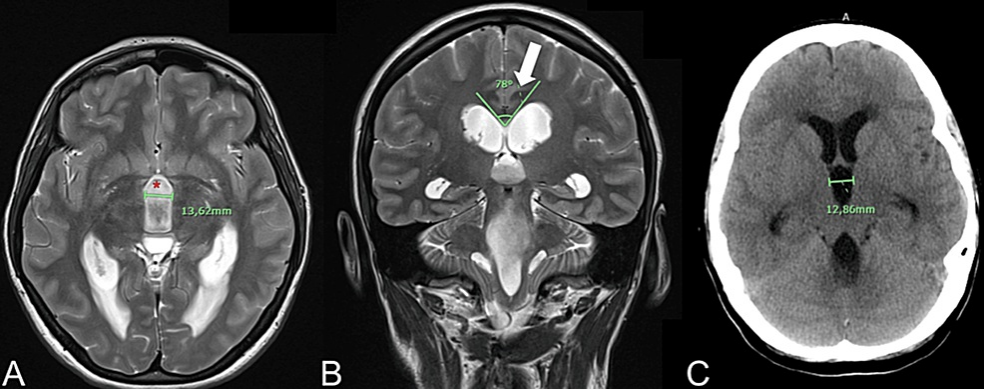
Radiological findings of Case 2 (A, B) MRI performed at the hospital admission: the axial and coronal images show ventriculomegaly with enlargement of third ventricle (red asterisk) and reduction of callosal angle (78°, white arrow). (C) Head CT scan performed at the time of neurological impairment, with negative COVID-19 test: no volume alteration of the ventricular system.

A 20-year-old unvaccinated female with a history of chronic migraine presented at our hospital complaining of a sudden worsening of the headache, unresponsive to her usual therapies, with concomitant nausea. At admission the patient was alert and oriented; neurological examination and vital signs were both normal. The molecular COVID-19 test was negative. Brain MRI showed dilated ventricles without CSF flow obstruction or space-occupying masses (Figures 2A, 2B). The patient was then scheduled for CSF depletive test.

The following day the patient presented a severe impairment of the neurological status with lethargy. The new CT scan was substantially unchanged compared to the admission images (Figure 2C). An urgent procedure of external ventricular drain (EVD) placement was performed, with an initial improvement of the neurological status. Two days after the admission, the routine COVID-19 molecular control test resulted positive. The subsequent period of isolation was uneventful without the necessity of special therapies. Once the molecular swab tested negative, the patient returned to our department and underwent a ventriculoperitoneal shunting procedure and was then discharged with complete resolution of her migraine.

## Discussion

Despite NPH has been first described almost 60 years ago [4], its pathophysiology still remains controversial and not completely clarified. In the last few years several studies focused on the role of inflammation mediators, such as cytokines and chemokines, in the pathogenesis of NPH [14-16]. It has been demonstrated, in fact, that the activation of NFKB and Toll-like receptor 4 inflammation pathway in the choroid plexus of rats induces CSF hypersecretion [10]. Nevertheless, different studies demonstrated high levels of other inflammatory mediators, in particular, TNF a [5,17,18], in the CSF of NPH patients, with a decrease in their level after shunt surgery [17,19]. Even if the role of inflammatory molecules in the alteration of CSF dynamics has not completely been revealed, these results suggest that an inflammation condition is present in the choroid plexus of NPH patients and may play a significant role in the genesis of hydrocephalus.

During COVID-19, the infected host develops a multifaceted inflammatory syndrome, that may lead to an uncontrolled immune system response [20]. CNS is recognized as a frequent target of SARS-CoV-2, due to a high representation of the Angiotensin Converting Enzyme 2 (ACE2) receptor that is a target of the virus Spike protein [21]. ACE2 receptor is known to be diffusely expressed in different areas of CNS, such as the olfactory epithelium, the temporal gyrus and posterior cingulate cortex and the choroid plexus [22,23]. In the infected choroid plexus, an alteration of its microscopical structure occurs, with syncytia formation, loss of integrity of the homeostatic barrier and activation of the cytokine production pathways [24,25]. As for idiopathic NPH, the massive release of inflammatory mediators [26,27] may result in the alteration of CSF dynamics and the consequent development of ventricle dilatation.

Considering these results, we believe that the sudden neurological impairment of our patients could be explained as a prodromal manifestation of SARS-CoV-2 infection. In fact, all our patients tested positive for molecular swabs the day after their clinical worsening, meaning that they were already infected by the virus. Even if we were not able to test CSF for COVID-19 antibodies due to laboratory limitations, the temporal succession of neurological impairment and diagnosis of SARS-CoV-2 infection, as well as the demonstration of a common inflammation condition in both diseases make, in our opinion, the hypothesis of correlation between the two events highly plausible. Moreover, the presence of anti-SARS-CoV-2 antibodies in the CSF of infected patients has been found in a study by Alexopoulos [28], supporting our hypothesis of an interaction between the inflammatory response triggered by the virus infection and the CSF environment. We can furthermore assume that the cephalalgia complained during COVID-19 infection may be a sign of an altered CSF dynamic.

To our knowledge there are no other cases reported in the literature of an NPH worsening shortly before the evidence of SARS-CoV-2 infection. Other authors reported cases of patients with “delayed” development of NPH after COVID-19 (one week three months from infection), requiring surgery for the treatment of the hydrocephalus [11-13]. All these patients did not have other risk factors for NPH or significant medical history apart from a recent SARS-CoV-2 infection. As for our article, the main limitation of these reports is undoubtedly their mainly observational and empiric nature.

## Conclusions

On the basis of our experience, we advise in NPH patients with sudden clinical worsening, to perform a molecular COVID-19 swab at the moment of clinical impairment and, in case of a negative result, to repeat the test in the following days. In addition to explaining the clinical worsening, significant benefits of an early diagnosis of COVID-19 (e.g., isolation of the patient, protection of the medical staff, activation of specific therapeutic protocols) are obtainable through widely accessible, reliable and low-cost molecular tests. We, therefore, recommend considering SARS-CoV-2 infection in the differential diagnosis of a sudden and otherwise unexplainable impairment of hydrocephalic patients. Furthermore, we believe clinicians should invite NPH patients to adopt adequate preventive measures to protect them from SARS-CoV-2 infection. Further studies, focusing on the molecular interaction between SARS-CoV-2, choroid plexus and CSF dynamics alteration, are needed to support our hypothesis.
